# Exploring the speed and performance of molecular replacement with *AMPLE* using *QUARK ab initio* protein models

**DOI:** 10.1107/S1399004714025784

**Published:** 2015-01-23

**Authors:** Ronan M. Keegan, Jaclyn Bibby, Jens Thomas, Dong Xu, Yang Zhang, Olga Mayans, Martyn D. Winn, Daniel J. Rigden

**Affiliations:** a Research Complex at Harwell, STFC Rutherford Appleton Laboratory, Didcot OX11 0FA, England; b Institute of Integrative Biology, University of Liverpool, Liverpool L69 7ZB, England; c Bioinformatics and Systems Biology Program, Sanford-Burnham Medical Research Institute, 10901 North Torrey Pines Road, La Jolla, CA 92037, USA; d Department of Computational Medicine and Bioinformatics, University of Michigan, Ann Arbor, MI 48109, USA; e Science and Technology Facilities Council Daresbury Laboratory, Warrington WA4 4AD, England

**Keywords:** *AMPLE*, *QUARK*, *ROSETTA*, *ab initio* modelling, molecular replacement

## Abstract

*AMPLE* clusters and truncates *ab initio* protein structure predictions, producing search models for molecular replacement. Here, an interesting degree of complementarity is shown between targets solved using the different *ab initio* modelling programs *QUARK* and *ROSETTA*. Search models derived from either program collectively solve almost all of the all-helical targets in the test set. Initial solutions produced by *Phaser* after only 5 min perform surprisingly well, improving the prospects for *in situ* structure solution by *AMPLE* during synchrotron visits. Taken together, the results show the potential for *AMPLE* to run more quickly and successfully solve more targets than previously suspected.

## Abstract 2

Two *ab initio* modelling programs solve complementary sets of targets, enhancing the success of *AMPLE* with small proteins.

## 1. Introduction   

Molecular replacement (MR) is by far the most popular route to the solution of the phase problem, accounting for over 70% of the structures deposited in the Protein Data Bank (PDB; Rose *et al.*, 2012 ▶) in recent years. In MR, phasing information is derived by placing a search model in the unit cell of the target to approximate its crystal lattice (Rossmann & Blow, 1962 ▶). The search model is typically an existing structure that is homologous and thus structurally resembles the target or an edited version thereof. Alternatively, homology modelling may produce an explicit prediction of the target structure for use as a search model. Either way, truly novel folds, that have not previously been structurally characterized, are generally rendered intractable for MR.

Key to broadening the applicability of MR is the exploitation of modelling approaches that can deal with targets whose folds are not, or are only poorly, represented in the PDB. One route is to model and place small fragments, such as isolated α-helices or characteristic motifs, whose local structure can be reliably predicted irrespective of the overall fold context (Rodríguez *et al.*, 2009 ▶, 2012 ▶; Sammito *et al.*, 2013 ▶). This approach, as implemented in *ARCIMBOLDO*, has achieved conspicuous successes but requires very significant computing resources. A second route is to use *ab initio* protein models as delivered by programs such as *ROSETTA* (Shortle *et al.*, 1998 ▶; Simons *et al.*, 1997 ▶, 1999 ▶), *I-TASSER* (Zhang, 2008 ▶; Roy *et al.*, 2010 ▶; Wu *et al.*, 2007 ▶) or *QUARK* (Xu & Zhang, 2012 ▶). These programs attempt to predict the entire structure of the target and generally function in two steps. Firstly, a rapid, fragment-assembly step operating on a reduced protein representation produces so-called ‘decoys’. Clusters of decoys represent candidate fold predictions which can then be subjected to a second step, an all-atom refinement which entails much greater CPU demands. All-atom *ab initio* predictions have succeeded in MR (Qian *et al.*, 2007 ▶; Das & Baker, 2009 ▶), but their computational needs prevent broader adoption. As an alternative, we have explored the use of the more quickly obtained decoys as search models. Using our method for decoy clustering and graded truncation, we showed that decoys solve more than 40% of a nonredundant set of small protein structures (Bibby *et al.*, 2012 ▶).

*Ab initio* methods for protein structure prediction are an active area of research, with iterative fragment-based approaches (Zhang & Skolnick, 2013 ▶) and new contact-based methods (Marks *et al.*, 2012 ▶) both pushing the size limit of tractable proteins. Here, we explore models produced by *QUARK* (Xu & Zhang, 2012 ▶), a new fragment-based approach that differs from *ROSETTA* in several important ways. For example, *QUARK* uses fragments of a size range of 1–20 residues, while *ROSETTA* typically employs only fragments of three or nine residues. Furthermore, the force field used differs, with that of *QUARK* combining both physical and knowledge-based energy terms. Finally, and of particular note, its novel collection of contacts based on distance profiles helps to pack the medium-to-long-range residue interactions (Xu & Zhang, 2013 ▶). Recent assessments have shown *QUARK* to be one of the best-performing methods in its class (Kinch *et al.*, 2011 ▶; Tai *et al.*, 2014 ▶). We find that *QUARK* solves an overlapping but distinctly complementary set of targets compared with previous work using *ROSETTA* (Bibby *et al.*, 2012 ▶). We also demonstrate that more recent versions of *Phaser* (McCoy *et al.*, 2005 ▶, 2007 ▶; Storoni *et al.*, 2004 ▶) and *SHELXE* (Sheldrick, 2008 ▶; Usón *et al.*, 2007 ▶; Thorn & Sheldrick, 2013 ▶), two key components of the *AMPLE* pipeline, produce significantly improved results. Finally, and unexpectedly, the imposition of a 5 min limit on *Phaser* degrades the success rate by less than 10%.

## 2. Materials and methods   

### 2.1. Materials   

For ease of comparison with previous results, we used our previously published set of 295 nonredundant protein targets (Bibby *et al.*, 2012 ▶; Supplementary Table S1). The selection criteria for these were a length of 40–120 residues, a resolution of better than 2.2 Å, an absence of bound metal or cofactor and *R* ≤ 0.25, *R*_free_ ≤ 0.35. They were grouped into three classes, all-α, all-β and mixed αβ, as described previously.

### 2.2. Methods   

For each sequence, *QUARK* (Xu & Zhang, 2012 ▶) produced 5000 individual structures. In the terminology of *ab initio* modelling, these low-resolution, rapidly obtained predictions are known as decoys. Briefly, the software first generated a set of structural fragments with lengths from one to 20 amino acids at each position of the query sequence. These fragments were used to assemble the *ab initio* models by replica-exchange Monte Carlo (REMC) simulations under the guide of a generic, atomic-level knowledge-based force field with consideration of various sequence-specific predicted structural features, including secondary-structure type, solvent accessibility and β-turn propensity. For each query, *QUARK* ran ten independent REMC simulations starting from different random numbers. In each run, 50 decoys were selected from each of the ten low-temperature trajectories, resulting in 5000 decoys. The decoys lacked the explicit side chains that a full, CPU-intensive modelling would add. PDB structures with a sequence identity of >30% to the target or detectable by *PSI-BLAST* (a criterion used by most of the *ab initio* folding benchmark tests; Zhang *et al.*, 2003 ▶; Simons *et al.*, 2001 ▶) were excluded from the fragment library.

Processing of decoys into search models used the *AMPLE* pipeline (Bibby *et al.*, 2012 ▶). Briefly, decoys were clustered using *SPICKER* (Zhang & Skolnick, 2004 ▶) and centroid structures representing the three largest clusters were used to generate ensembles containing structural neighbours. Side chains were added to the ensembles using *SCRWL* (Canutescu *et al.*, 2003 ▶; Krivov *et al.*, 2009 ▶). The structural diversity within each ensemble predicts the deviation from the true structure (Qian *et al.*, 2007 ▶; Bibby *et al.*, 2012 ▶), and therefore the variance along the chain was quantified with *THESEUS* (Theobald & Wuttke, 2006 ▶) and used to derive up to 20 progressively more truncated versions of each ensemble. A sub-clustering step, collecting up to 30 near-centroid structures at 1, 2 and 3 Å r.m.s.d. thresholds, produced more structurally homogeneous ensembles from these results. After treatment of side chains in three different ways (all retained; only more easily predicted side chains retained and others trimmed to polyalanine; all trimmed to polyalanine) these subclusters became the set of search models. Hundreds of distinct search models may be produced for a single target. They are dealt with by *MrBUMP* (Keegan & Winn, 2008 ▶). In this work, only *Phaser* (McCoy *et al.*, 2005 ▶, 2007 ▶; Storoni *et al.*, 2004 ▶) was used for MR solution. Automated density modification and main-chain tracing with *SHELXE* (Sheldrick, 2008 ▶; Usón *et al.*, 2007 ▶; Thorn & Sheldrick, 2013 ▶) was used to detect successful solutions as having a CC value of ≥25 and a mean traced chain-fragment length of ≥10. For *Phaser*, default parameters were used with the exception of the estimated r.m.s.d. error (see below). For *SHELXE*, the following options were used: 15 cycles of autotracing (-a15), searching for α-helices (-q), pruning for optimization of the CC for the input model (-o), the time factor for the helix and peptide search (-t3) and the ‘free-lunch’ option to add missing data up to 1.0 Å resolution if the data resolution was 1.8 Å or better (-e1.0). All other options were set to their default values. Mean phase error (MPE) values were calculated using *CPHASEMATCH* from the *CCP*4 suite (Winn *et al.*, 2011 ▶). Here, focusing on overall success rates, *AMPLE* terminated after finding the first success.

For comparison with published data, *QUARK*-derived search models were run with *Phaser* 2.3.0 and *SHELXE* 2012 (Run 1). The estimated r.m.s.d. error of the search models was set to 0.1 Å, as previously (Bibby *et al.*, 2012 ▶), or to 1.2 Å (Run 2 alone). *QUARK*-derived search models were also run with *Phaser* 2.5.4 and *SHELXE* 2013 (Run 3). Since MR is typically the slowest step in* AMPLE*, a requested time limit of 5 min for *Phaser* (KILL TIME 300 flag) was also tested (Run 4). In practice, since *Phaser* is only terminated at certain points in the code, this most commonly stops Phaser after 10–20 min.

## 3. Results and discussion   

### 3.1. Overall performance of *QUARK* models   

**Figure 1:**
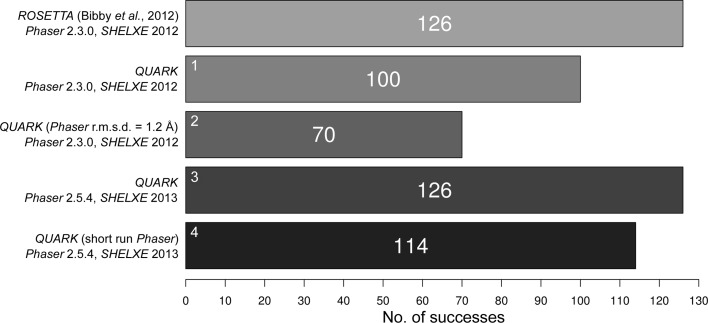
Numbers of targets solved with *QUARK*-derived search models under various conditions compared with previous results (Bibby *et al.*, 2012 ▶). The small numbers at the top left of the columns indicate the run numbers referred to in the text.

*ROSETTA*-derived search models processed with *Phaser* previously solved 126 of the 295 targets. The result for the *QUARK* set, using the same *Phaser* and *SHELXE* versions and operating parameters, is 100/295 (Run 1). As previously (Bibby *et al.*, 2012 ▶), when producing the *QUARK* models homologous fragments were excluded to treat each target as if it were a novel fold. For comparison we also tested providing *Phaser* with a 1.2 Å estimated r.m.s. error in the search models (Run 2), as opposed to the 0.1 Å value employed in Run 1. This dramatically reduced the success rate to 70/295 and thus the value of 0.1 Å was used for all of the remaining runs. We then tested the success of the *QUARK* models using the latest versions of *Phaser* and *SHELXE* (Run 3) and found a steep increase in success to 126 of the 295 cases (Fig. 1 ▶).

**Figure 2:**
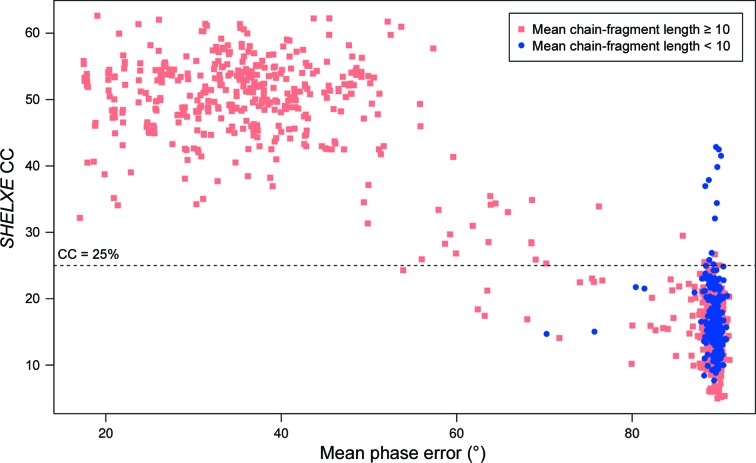
Comparison of *SHELXE* CC and mean phase error for Runs 1–4 combined. Each point represents a search model and the values are either those of the successful solution or those of the highest failing CC score. Symbols distinguish *SHELXE* traces that do or do not exceed a mean traced chain-fragment length of 10. In all cases 15 cycles of auto-tracing were invoked in *SHELXE*. Each cycle included 20 iterations of density modification.

In this work, success is defined by *SHELXE* (Sheldrick, 2008 ▶; Usón *et al.*, 2007 ▶; Thorn & Sheldrick, 2013 ▶) criteria: a CC value of ≥25 and a mean traced chain-fragment length of ≥10. The work of Thorn & Sheldrick (2013 ▶) suggested that a CC of ≥25 and native data to better than 2.5 Å resolution are invariably indicative of success. Since we are benchmarking against known crystal structures, mean phase errors (MPEs) for successful and failing search models can be calculated (Fig. 2 ▶). The vast majority of the cases defined as successful by the *SHELXE* criteria indeed have a low MPE. However, in a handful of cases, totalling only seven search models across all of Runs 1–4, solutions classified as successful have an MPE of >75°. Four of these seven false positives relate to PDB entry 2fu2 which, although reported to have a resolution limit of 2.1 Å, diffracted anisotropically to only 2.6 Å in the worst direction. This potentially explains the poor quality of the solutions despite the *SHELXE* statistics. PDB entry 2qyw (twice) and one search model for PDB entry 3n3f gave the other false positives, and no obvious explanation for the failure of the criteria in these cases is evident. However, three such cases in a set of 1117 (Fig. 2 ▶) is a very low failure rate of the *SHELXE*-based success criteria and we note that these three cases only marginally passed either the CC or the mean traced chain-length criteria.

The MPE plot (Fig. 2 ▶) also reveals several targets that have a relatively low MPE but failed to meet the *SHELXE* scoring criteria. Several cases were examined in more detail: PDB entries 1vjk (MPE = 63.2°) and 2rff (62.4°) from Run 1, 3oiz (63.5°) from Run 3 and 1xak (70°) from Run 4. Of these, 1vjk and 3oiz could be easily improved upon through further cycles of *SHELXE*, achieving MPE values of 28.7° (CC = 36.1, average chain length = 71) and 36.2° (CC = 47.6, average chain length = 46.5), respectively. The MPE for 2rff and 1vjk could not be improved by further cycles of *SHELXE*. However, in both cases the partial C^α^ trace produced by *SHELXE* was correct when compared with the deposited structure for these targets using the *CSYMMATCH* program from the *CCP*4 suite.

**Figure 3:**
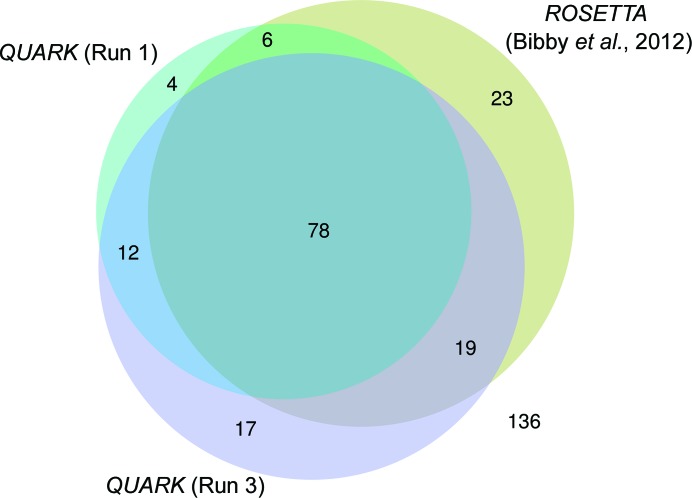
Venn diagram illustrating successful solutions using *QUARK*-derived search models (Runs 1 and 3) compared with previous results (Bibby *et al.*, 2012 ▶).

The significant methodological differences between *ROSETTA* and *QUARK* led us to assess their performance across target classes and, thereby, their complementarity to increase the overall success rate of *AMPLE* (Fig. 3 ▶). Using the same versions of *Phaser* and *SHELXE*, 16 *QUARK* (Run 1) and 42 *ROSETTA* successes were uniquely achieved by each program. Jointly, *ROSETTA* and *QUARK* successes amounted to 142 targets (48% of the total, compared with 43% reported for *ROSETTA* alone). Interestingly, the same *QUARK*-derived search models were significantly more successful with more recent versions of *Phaser* and *SHELXE* (Run 3), solving 29 targets that were not previously solved with *ROSETTA*. This illustrates how the *AMPLE* pipeline continuously combines advances in various methodologies to deliver the best performance for automated MR. All runs considered, *AMPLE* solved 54% of targets, up from 43% with only *ROSETTA* models (Bibby *et al.*, 2012 ▶).

**Figure 4:**
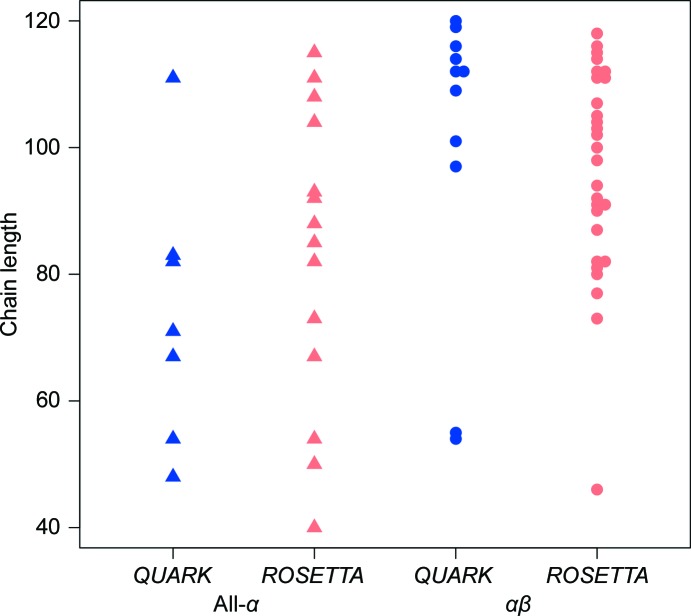
Chain lengths and secondary-structure classes of targets uniquely solved using either *QUARK*-derived (Run 1) or *ROSETTA*-derived search models (18 and 42 cases, respectively; Bibby *et al.*, 2012 ▶).

The search models from *QUARK* predictions performed similarly across secondary-structure classes as the *ROSETTA*-derived search models (Fig. 4 ▶). In our test set there are 77 all-α, 44 all-β and 174 αβ targets. Particularly noticeable is the poor performance of both programs with all-β targets. The two all-β targets previously solved were also solved by a *QUARK*-derived search model, but no further successes were added. In contrast, 60 all-α targets solved previously with *Phaser* (Bibby *et al.*, 2012 ▶) were complemented by four additional successes from *QUARK* (Run 1), taking the success rate between the two runs to 83%. Remarkably, including Run 3, with modern versions of *Phaser* and *SHELXE*, adds a further seven targets solved at least once between Runs 1 and 3 here and previous results (Bibby *et al.*, 2012 ▶): thus, very nearly all of the all-α targets in the set (92%) were solved at least once. In the αβ class, it is notable how the complementarity with *QUARK* is focused in the larger target-size range above 100 residues or so. As previously (Bibby *et al.*, 2012 ▶), success close to the upper size limit should encourage the application of *AMPLE* to larger targets.

Structure solution was achieved across a broad range of diffraction resolutions in the test set. The lowest resolution success was at 2.1 Å (PDB entry 3kw6). The highest resolution target (PDB entry 1ejg, 0.54 Å) was solved in both the original *ROSETTA* run and in Run 3 here. In general, low solvent content corresponds to higher resolution, and in our tests in all runs the solved structures had a solvent-content fraction ranging from 10.5 to 70.8%. The mean solvent contents for the sets of solutions solved by *ROSETTA* and each of the *QUARK* run solutions were around 46%, compared with a mean solvent content in the whole target set of 45%. Thus, in the ranges explored, resolution and solvent content do not appear to have been an influence on solubility in our test set when using *ROSETTA*-derived or *QUARK*-derived models. However, a more extensive set of tests over a wider range of resolutions would be needed in order to obtain a clearer picture of the relevance or otherwise of these factors.

12 targets in the test set contained two molecules per asymmetric unit and the rest contained a single molecule. Interestingly, Run 3 was the most successful for these targets, solving 11 of the 12 targets; *ROSETTA*, Run 1 and Run 4 could solve eight, while Run 2 could solve seven. This suggests that use of the latest versions of *Phaser* and *SHELXE* may be particularly important in these cases.

### 3.2. Results from faster *Phaser* runs   

The MR step is computationally demanding in *AMPLE*, accounting for around 33% of the runtime on average. For successful cases in Run 3, runtimes averaged about 48 h per target with pre-calculated *QUARK* models so, although *AMPLE* allows parallelization on clusters and multi-core machines, there is nevertheless an incentive to explore ways of speeding up its operation. *Phaser* regularly outputs its current best result during operation and the KILL TIME flag allows runs to be terminated after a user-specified time. The results show that requesting that *Phaser* limit the CPU time to 5 min (in practice, up to 30 min; see §[Sec sec2]2) has a surprisingly small impact on performance (Fig. 1 ▶), with the successfully solved targets only decreasing from 125 to 114. Interestingly, only 103 targets are shared between the long and short *Phaser* results (Runs 3 and 4, respectively). Supplementary Fig. S1 shows no obvious systematic differences in the characteristics of targets solved exclusively in the shorter run although, unexpectedly, two all-β targets, which are generally harder to solve, were among them. The average time spent running *Phaser* in successful cases in Run 3 is approximately 16 h per target, with an average of 18 search models tested before a solution is found. In Run 4, the average time spent running *Phaser* in successful cases drops to 5 h per target, with an average of 25 search models tested before a solution is found. Thus, although more search models have to be tested in the short *Phaser* run, on average a similar overall per-target success rate is achieved in a distinctly shorter time. More elegant options for limiting *Phaser* runtimes based on restricting the number of trial orientations or solutions will be explored in future work.

## 4. Concluding remarks   

The exploitation of unconventional sources of search models is an appealing route to enhancing the applicability of the MR method for structure solution. Given the increased rates of protein crystallization provided by nanodispensing instrumentation and the accelerated speed of data collection at modern synchrotrons, the demand for purely computational phasing approaches that can offer full automation is becoming ever more pressing. These results show that different *ab initio* methods are complementary in terms of the targets solved in our benchmarking set. Between earlier results and the comparable *QUARK* results presented here (both Runs 1 and 3 combined), 159 of 295 targets (54%) were solved. Although differing in the software used, it is remarkable that 93% of the all-α targets in the set are demonstrably soluble using either *ROSETTA*-derived models (Bibby *et al.*, 2012 ▶) or *QUARK* models (Runs 1 or 3). Thus, almost all small helical proteins can potentially be solved using *ab initio* models from the current generation of modelling software. Contrariwise, the disappointing results for all-β targets using both *ROSETTA* and *QUARK* (5%) suggest there is still a need for significant improvements to *ab initio* methods for this class of targets. The time-limited *Phaser* results suggest that an impatient *AMPLE* user may achieve good results in a shorter time than previously envisaged. Such quick *AMPLE* runs could, for example, aid *in situ* structure solution of new folds during diffraction data collection at synchrotrons.

The complementarity of targets solved with *ROSETTA* or *QUARK* shows that, computational resources allowing, these programs should be used jointly. *AMPLE*’s approach requires access to the set of decoy structures rather than the selected fold predictions currently available from some *ab initio* modelling servers. Currently, *ROSETTA* is distributed for local use and *QUARK* is freely available for online server submission, with results including both final models and trajectories of folding decoys. Use of the server eliminates the requirement for *AMPLE* MR of a local installation of *ab initio* modelling software. *QUARK* decoys can be used in *AMPLE* from the command line using the ‘-quark_models’ flag. Alternatively, the *CCP*4*i* interface for *AMPLE* provides a link to the *QUARK* server, where users can generate the decoys.tar.gz file that is subsequently employed for search-model generation and MR. A local version of *QUARK* should become available in the near future.

## Supplementary Material

Supplementary Figure S1.. DOI: 10.1107/S1399004714025784/rr5080sup1.pdf

Supplementary Table S1.. DOI: 10.1107/S1399004714025784/rr5080sup2.xlsx
