# Supplementary material for: Exploring the speed and performance of molecular replacement with AMPLE using QUARK ab initio protein models
Source: Acta Crystallogr D Biol Crystallogr. 2015 Jan 23;71(Pt 2):338–43. doi: 10.1107/S1399004714025784 (PMC4321487; doi:10.1107/S1399004714025784)
Supplement: Supplementary file 1 [file d-71-00338-sup1.pdf]

Acta Cryst D
BIOLOGICAL CRYSTALLOGRAPHY

**Volume 71 (2015)**

**Supporting information for article:**

# Exploring the speed and performance of molecular replacement with *AMPLE* using *QUARK ab initio* protein models

**Ronan M. Keegan, Jaclyn Bibby, Jens Thomas, Dong Xu, Yang Zhang, Olga Mayans, Martyn D. Winn and Daniel J. Rigden**

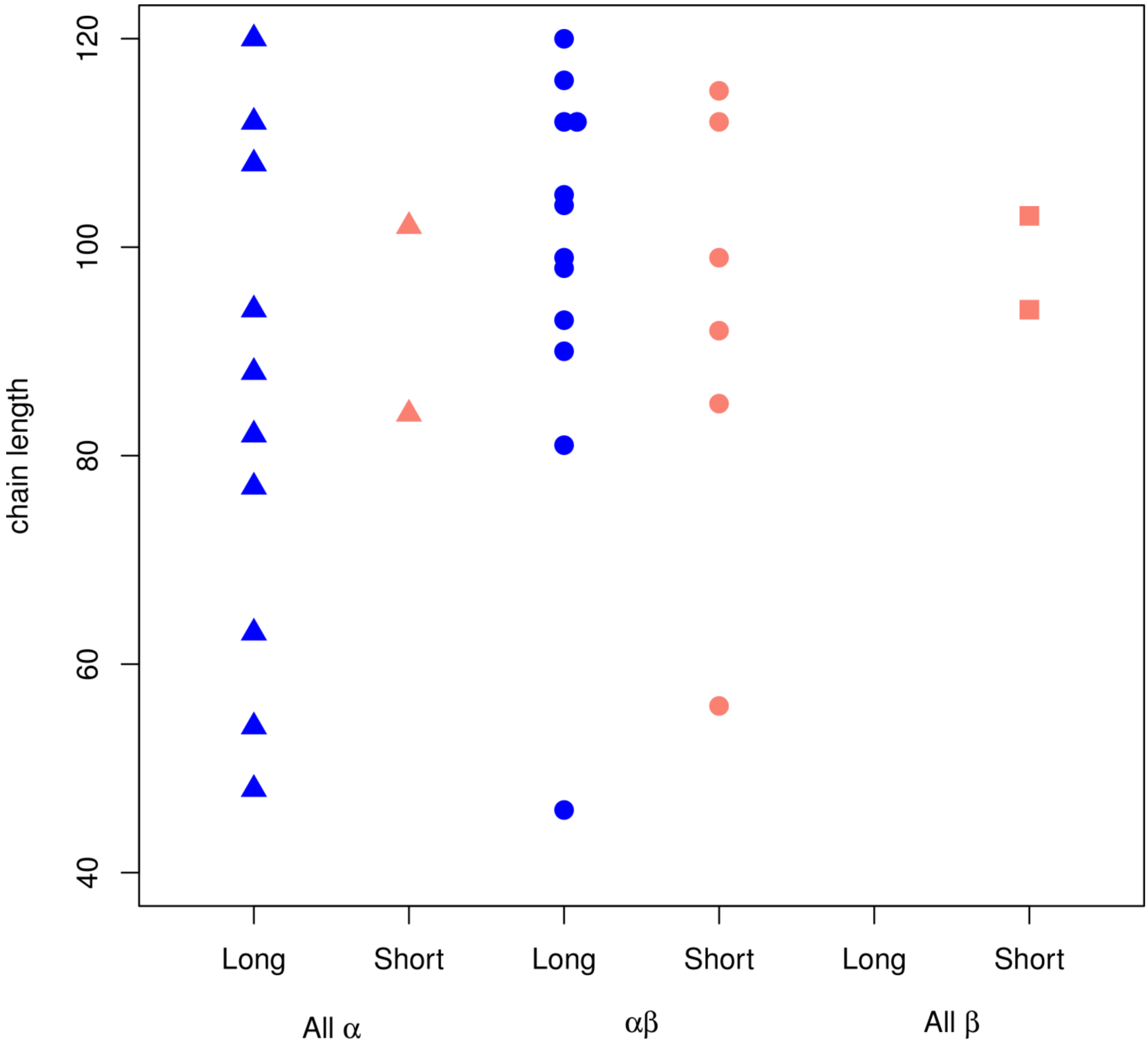


**Figure S1** Secondary structure class and length of targets solved in either Run 3 (‘Long’ up to 12 hours Phaser) or Run 4 (‘Short’ 5 minutes Phaser) but not in the other.
